# Evolutionary tools for phytosanitary risk analysis: phylogenetic signal as a predictor of host range of plant pests and pathogens

**DOI:** 10.1111/j.1752-4571.2012.00265.x

**Published:** 2012-05-03

**Authors:** Gregory S Gilbert, Roger Magarey, Karl Suiter, Campbell O Webb

**Affiliations:** 1 Environmental Studies Department, University of CaliforniaSanta Cruz, CA, USA; 2 Smithsonian Tropical Research InstituteBalboa, Ancón, Panama; 3 Center for Integrated Pest Management, North Carolina State UniversityRaleigh, NC, USA; 4 Arnold Arboretum of Harvard UniversityBoston, MA, USA

**Keywords:** emergent pests and pathogens, phylogenetic ecology, plant disease ecology, fungal pathogens, herbivory, novel species interactions, biological invasions

## Abstract

Assessing risk from a novel pest or pathogen requires knowing which local plant species are susceptible. Empirical data on the local host range of novel pests are usually lacking, but we know that some pests are more likely to attack closely related plant species than species separated by greater evolutionary distance. We use the Global Pest and Disease Database, an internal database maintained by the United States Department of Agriculture Animal and Plant Health Inspection Service – Plant Protection and Quarantine Division (USDA APHIS-PPQ), to evaluate the strength of the phylogenetic signal in host range for nine major groups of plant pests and pathogens. Eight of nine groups showed significant phylogenetic signal in host range. Additionally, pests and pathogens with more known hosts attacked a phylogenetically broader range of hosts. This suggests that easily obtained data – the number of known hosts and the phylogenetic distance between known hosts and other species of interest – can be used to predict *which* plant species are likely to be susceptible to a particular pest. This can facilitate rapid assessment of risk from novel pests and pathogens when empirical host range data are not yet available and guide efficient collection of empirical data for risk evaluation.

## Introduction

Novel interactions between plants and pests or pathogens pose economic and ecological threats to agricultural and wildland ecosystems (Pimentel et al. 2000). Novel pest–plant interactions (‘pest’ used here collectively to mean natural enemies of plants, be they microbes, animals, plants, viruses, etc.) emerge when humans introduce plant pests accidentally (e.g., through trade) (Goodell et al. 2000) or purposefully (e.g., biocontrol) (Barton 2004); when pests arrive autonomously to a new region through range expansion (e.g., facilitated by climate change) (Anderson et al. 2004); or when novel pests evolve *in situ* (e.g., through hybridization) (Brasier 2001). The task of governmental phytosanitary agencies is to ensure national or regional plant biosecurity through an array of preventative and management activities such as quarantine, port-of-entry interception, eradication, and control of novel pests (Magarey et al. 2009). But not all novel pests are significant threats (Parker et al. 1999), and because prevention and management efforts incur significant economic and political costs, pest risk assessment is essential to determine appropriate actions. Robust analytical tools, based on sound scientific understanding of plant–pest relationships, are critical to help evaluate which pests and pathogens represent risks that warrant action (Campbell 2001; Briese 2003; Magarey et al. 2009). Here, we show how the evolutionary structure of host ranges of plant pests can provide the basis for a useful new tool in pest risk analysis when empirical data are limiting, allowing a rapid assessment of *which* plant species in an area are most likely to be susceptible to a novel pest.

**Figure 1:**
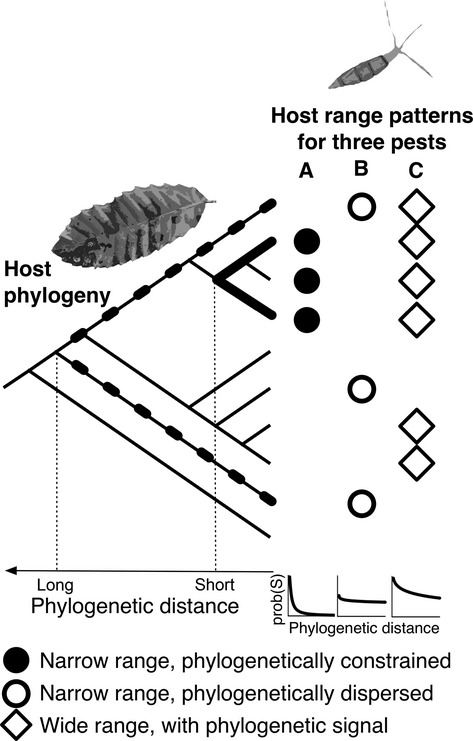
Expected phylogenetic patterns in host ranges of plant pests and pathogens. Phylogenetic signal in the host range of pests is a measure of the probability that two hosts (two branch tips on the phylogenetic tree) would share a particular pest, given the phylogenetic distance between the hosts. (A) The host ranges of most pests and pathogens are expected to show a phylogenetic signal, with a tendency to be clustered within an evolutionary clade (filled circles). The probability of sharing a pest (prob(S)) decreases quickly with increasing phylogenetic distance between the hosts. (B) Phylogenetically dispersed host ranges (open circles) should be less common, because phylogenetically dispersed hosts should present diverse defensive traits that would be challenging for a single pest to overcome. (C) Pests with broad host ranges are likely to show a phylogenetic signal (open diamonds), where expanded host range comes with the ability to attack several hosts within multiple clades. Phylogenetic distance is measured as time of independent evolution between two taxa, represented here by the thick solid line (short phylogenetic distance) and thick dashed line (long distance). Time of independent evolution is twice the age to most recent common ancestor (vertical dotted lines).

Plant pests and pathogens are often able to attack a number of closely related species (Gilbert and Webb [Bibr b20]). Interactions between plants and their pests and pathogens are governed strongly by the presence (or absence) of a variety of plant chemical, morphological, and life-history traits (Herms and Mattson 1992; Coley and Barone 2011; Bradley et al. 2003; Carmona et al. [Bibr b13]). Such traits are often phylogenetically conserved – closely related plants have more similar suites of traits that are important to pest interactions than do more evolutionarily distant plant species (Fluhr and Kaplan-Levy 2002; Parker and Gilbert 2004; Agrawal 2007; Boller and Felix 2009; Pearse and Hipp [Bibr b34]). This generates a phylogenetic signal in host ranges, where closely related plant species should be more likely to share a particular pathogen or pest, than should distant relatives (Fig. 1). How close, then, is close enough for two hosts to share a pathogen or pest? Is the evolutionary structure in host ranges a step function – pests can attack many species within a genus, but not beyond – or does the probability of sharing hosts decline continuously as a function of evolutionary distance?

Empirical studies support the expectation of a phylogenetic signal in host ranges of fungal plant pathogens. Cross-inoculations across many plant species with foliar pathogens show that most fungal pathogens are polyphagous (i.e., attack multiple hosts) and that the likelihood that a pathogen can cause disease on two particular plant species is greatest among congeneric species, strong within plant families, and declines gradually even to the most ancient evolutionary distances within flowering plants (Gilbert and Webb [Bibr b20]). Similar patterns of phylogenetic signal in host range are evident for herbivorous insects (Novotny et al. 2002; Weiblen et al. 2006; Pearse and Hipp [Bibr b34]) and pathogens of animals (Pedersen and Davies 2009). In fact, although there are clear examples of where the distribution of traits that are important to interactions with natural enemies can be independent of phylogeny (e.g., Becerra and Venable 1999), phylogenetic conservatism is common for ecological interactions of all types across the entire tree of life (Gómez et al. 2007). Phylogenetic distance between potential host species thus appears to be a good, integrated surrogate for the differences in plant traits that determine the host ranges of plant pests and pathogens. It is also much easier, faster, and cheaper to use existing dated phylogenetic supertrees (large phylogenies constructed from multiple source phylogenies) (Davies et al. 2004) to quickly estimate phylogenetic distance among plant species (Webb and Donoghue 2005) than it is to determine which plant traits are important to a particular pathogen, or to empirically test a large number of hosts for susceptibility to a novel pathogen. If this continuous phylogenetic signal is robust across the broad range of plant pests (e.g., bacteria, invertebrates, parasitic plants, viruses), it suggest an important new tool for pest risk assessment novel plant pests, as well as a useful framework for ecological researchers studying species interactions. However, evaluating the utility of this phylogenetic signal first requires examination of phylogenetic signal in host range across a broad diversity of hosts and pest types.

The United Stated Department of Agriculture has systematically gathered global records of occurrence of pests and pathogens on plants for many decades (Magarey et al. 2009). The United States Department of Agriculture (USDA) databases, as well as similar compendia produced by CABI, are used globally by governmental agencies and agronomists to make phytosanitary decisions, including those that affect trade, quarantine, and eradication efforts. The databases are also used for ecological research. A publicly accessible database of Fungus-Host Distributions maintained by the USDA Agricultural Research Service (Farr et al. 2007), based primarily on published records of fungi on plants and plant products, has been used for a number of studies on the ecology of plant diseases (e.g., Mitchell and Power 2003; van Kleunen and Fischer 2009). The USDA Animal and Plant Health Inspection Service – Plant Protection and Quarantine Division (APHIS-PPQ) maintains the Global Pest and Disease Database (GPDD) for exotic pest risk analysis (Magarey et al. 2009). Initiated in 2003, the GPDD is a compendium of over 3100 pest species of agronomic importance that currently are either not present in or are restricted in their United States distribution. This database contains approximately 137 000 pest-host records on over 18 500 plant species from around the globe. Data are gathered from publications, more than 300 compiled lists and databases, border interceptions, pest surveys, pest and commodity risk assessments, and publicly available source material. Data from the GPDD are critical to decisions that affect trade regulation, quarantine, and eradication activities. Nevertheless, like all such databases, the GPDD includes significant assumptions and limitations. Use of the data from the GPDD in this study was arranged by means of a contractual cooperative agreement between G. Gilbert, North Carolina State University and APHIS-PPQ-CHPST.

The inherent assumptions and limitations of host range databases like the Fungus-Host Distributions database and the GPDD could systematically bias analytical results for studies of host range of pests and the number of pests on particular hosts. Limitations include survey bias, informatics bias, and structural bias. Survey bias means that pest surveys that form the basis for publication and interception records have a strong bias toward plants important in agriculture, horticulture, and forestry in regions with many active researchers in phytosanitary and research institutions and have much more limited coverage of wildlands species. As such, knowledge of pests of environmental hosts or crops of limited regional importance are unlikely to be complete. Analyses that use the number of recorded hosts or the diversity of recorded pests in the database as indications of pest impacts (Mitchell and Power 2003; van Kleunen and Fischer 2009) are particularly vulnerable to systematic biases. Some researchers have tried to correct for this bias by using an index of effort of investigation [e.g., standardized by the number of publications on a particular plant species (Gibson et al. 2010)]. Informatics bias results from unstable and inconsistent taxonomy (e.g., anamorph and teleomorph names for ascomycete fungi), unverified identifications, and geographic variation in coevolutionary dynamics (i.e., genotypic variation in virulence/resistance). Careful use of pest nomenclature, as well as maintenance of current taxonomic synonymies, is essential to avoid exaggerated apparent diversity of pests on a single host (when multiple names are used for the same entity) or exaggerated host ranges (when a single pest name is applied to several cryptic biological entities). For example, queries to the USDA Fungal-Host Distributions database produce large numbers of synonyms that appear as separate pests, requiring diligent data corrections on the part of the user. Finally, host range databases have a structural bias because they include only records of incidence of a pest or pathogen on a plant host and do not record the *absence* of a pest on a particular host. This is particularly problematic for pests and plants that are geographically restricted (such as emerging pests or endemic hosts); geography may have so far prevented a pathogen from being observed on a plant on a different continent, but that host may still be susceptible. Because these databases lack specific data on which hosts are *not* susceptible, analyses of host range based on the databases require an explicit assumption that the ‘zeroes’ are true, which likely leads to many false negatives. Estimates of pest diversity and host range from such databases are thus likely to be lower bounds.

By definition, there are few antecedents to help evaluate which local plants species are susceptible to a novel plant pest. Widespread agricultural crop species (e.g., rice, beans) are likely to have robust pest records, but there will usually be little information on which regional crops or native environmental plant species are susceptible in the region of introduction. When a pest or pathogen is introduced intentionally into a new region (e.g., for biological control of an invasive weed) or arrives without human intent, prior knowledge and rapid assessment of locally susceptible hosts is imperative. Empirical host range testing (e.g., Weidemann and Tebeest 1990; Gilbert and Webb [Bibr b20]) is slow, expensive, and impractical at the scale necessary in a rapidly changing, globalized world unless there is clear guidance for prioritizing likely hosts. In the absence of existing empirical data, phytosanitary agencies have developed some phylogenetic rules-of-thumb: a pest may be considered a threat if it is known from a plant species in the same genus as a local species of concern (APHIS 2005); concern about host range is ranked as low if the pest attacks species within a single genus, medium if the pest is limited to a single family, and high if it attacks multiple families (PPQ 2003); which plant species are selected for host range testing of pathogens being considered for release as biological control agents often follows a ‘centrifugal phylogenetic method’ (Wapshere 1974; Briese 2003). However, more robust analytical tools that can quickly and inexpensively provide information on *which* local plant species are likely to be hosts for a particular novel pest would better guide phytosanitary risk analysis and direct efficient use of subsequent empirical tests.

Here, we use host range data from the USDA APHIS-PPQ GPDD to measure the strength of a phylogenetic signal in host range across each of nine major groups of plant pests and pathogens and to evaluate the potential for evolutionary tools to inform pest risk analyses. First, we explore the pests' host-breadth structure as recorded in the APHIS-PPQ GPDD. Next, we test for phylogenetic signal in host range within each of the major groups of pests and pathogens and compare the phylogenetic signal to that determined empirically for fungal foliar pathogens, to evaluate whether the analysis is robust to assumptions required because of the structural bias in host range databases (i.e., no nonhost records). Third, we evaluate how the known number of hosts of a pest interacts with phylogenetic signal to provide a more robust estimate of the likelihood of sharing across particular hosts. Finally, we suggest how this understanding of the evolutionary structure of host range can help evaluate which local plants are most likely to be hosts for a novel pest or pathogen.

## Materials and methods

We based our analyses on data taken from the USDA APHIS-PPQ GPDD (APHIS-PPQ GPDD). We extracted all recorded plant pests from 210 genera of flowering plants. Pests were classified to species or to finer levels if appropriate (e.g., pathovars of bacteria). Host plants were grouped to genus; if a pest occurs on any species in a genus, that genus was considered susceptible. Each pest species/host genus combination was scored as 1 (host is susceptible) or 0 (host is assumed to be resistant). Grouping to genus provides us with a conservative test of phylogenetic signal, given that it is well established that pests and pathogens are likely to be able to attack multiple species within a genus (Novotny and Basset 2005; Farr et al. 2007). In addition, taxonomic uncertainty for plant species for host records at a global level, the poor coverage of sampling across many species within a genus, and the lack of readily accessible phylogenetic trees within many plant genera all limit the utility analyses at the level of host species. At the same time, grouping pest records within a genus provides the maximum information about phylogenetic signal at farther phylogenetic distances, where host ranges are less well known.

We took a re-sampling approach to the analysis of the APHIS-PPQ GPDD data to (i) make the analysis comparable to the empirical work on phylogenetic host range in plant pathogenic fungi established by Gilbert and Webb ([Bibr b20]), (ii) reflect the practical situation of finding a novel pathogen on a single plant host, and (iii) avoid problems with inflated degrees of freedom and pseudoreplication, because the probability that a pest found on plant A also occurs on plant B is not independent of the likelihood that a pest from plant B also attacks plant A.

Specifically, for each pest species, we randomly selected one host genus from among the hosts on which the pest had been reported, and assigned it as the ‘source’ host. For each of the other 209 ‘target’ plant genera in the database, we then recorded whether the genus was listed in the database as susceptible or not and the phylogenetic distance from source to target genus. We also recorded the total number of known host genera in the database for that pest species. This was repeated for all pest species, using one randomly selected source genus per pest.

We then analyzed those data using logistic regression, where the response variable was 1 (susceptible) or 0 (assumed resistant). For the first logistic analysis, which parallels the analysis of empirical data from Gilbert and Webb ([Bibr b20]), the independent variable was the phylogenetic distance between source and target plant species [transformed as log_10_(phylodistance + 1)]. For the subsequent logistic analysis, we included the phylogenetic distance term and the number of known hosts for that pest. The interaction term was not included in final models, because it was not significant (see Results). Intercept and slope coefficients were recorded. This was repeated for 1000 total runs, with new random selections of source plant hosts for each pest in each run, and recording all 1000 sets of coefficients. We calculated the median intercept and slope coefficients and the 95% confidence interval across all runs. If the 95% confidence interval of a coefficient did not include zero, it was considered significant. To illustrate the confidence intervals graphically, we also captured predicted values of the probability of sharing a pathogen at 5-My intervals, for each of the 1000 models, and then calculated the 0.025, 0.5, and 0.975 quantiles to use for 95% confidence intervals of the curves.

This basic approach was used separately for each of nine biologically meaningful groups of pest and pathogens: bacteria, fungi, oomycetes, insects, mites, mollusks, nematodes, viruses, and plants. These divisions were determined based on pests and pathogens belonging to different kingdoms (bacteria, fungi, oomycetes, plants, viruses, and animals) and very different life-history and feeding strategies (insects, mites, mollusks, and nematodes within the animals). Further refinements in analysis of variation across life-history strategies are left for future work.

Phylogenetic distances were calculated from the APG II supertree of Davies et al. (2004), which included dated nodes given by Wikstrom (Wikstrom et al. 2001). This version was used to be consistent with earlier empirical work, but using more recent structure of APG III et al. (2009) should have little effect on the model. We used Phylomatic version included in Phylocom v4.1 (Webb et al. #b^501^) to create a pruned ultrametric tree of all 210 Angiosperm genera in the database, with branch lengths that reflected the estimated time between branching events. From this tree, we used the phydist function in the R package Picante (Kembel et al. #b^500^) to calculate pairwise phylogenetic distances in My for each pair of plant host genera (given as time of independent evolution, which is twice the time to most recent common ancestor).

Analyses were completed using R statistical framework, with functions from the Picante v. 1.2-0 (http://cran.r-project.org/), Vegan v. 1.17-8 (http://cran.r-project.org/), and Stats v. 2.12.2 (http://cran.r-project.org/)packages.

## Results

### Structure of host ranges and pest diversity in the GPDD

The subset of the GPDD data used in this analysis comprised 1670 pest species and 210 host angiosperm genera (Table S1). Pest species included 52 bacteria, 212 fungi, 61 oomycetes, 870 insects, 119 mites, 45 mollusks, 105 nematodes, 71 parasitic plants, and 135 viruses (Table S2). Of the 350 700 possible host–pest combinations in the database, 15 328 (4.37%) were confirmed host records for a pest; we assumed (conservatively) that the rest were incompatible interactions.

**Figure 2:**
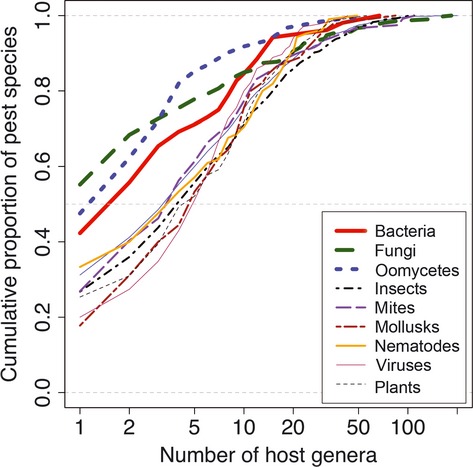
Number of known hosts for pests in each of each of nine major groups. The empirical cumulative distributions for number of known hosts for bacteria, fungi, and oomycetes (microbial pathogens) do not differ significantly from each other (K-S test, *P* > 0.08 for all), and the distributions for insects, mites, mollusks, nematodes, viruses, and plants (pests + viruses) do not differ significantly from each other (*P* > 0.15 for all). The microbial pathogen distributions differ significantly from all the pests+virus distributions (*P* < 0.035 for all) except for bacteria versus mites, mollusks, and nematodes (*P* > 0.08). Details given in Fig. S1.

The microbial pathogens (bacteria, fungi, and oomycetes) showed narrower median host ranges than did pests and viruses (insects, mites, mollusks, nematodes, plants, and viruses) (Figs 2 and S1, Tables S3 and S4). Of the microbial pathogens, about half (42–55%) were recorded from a single host genus (median number of hosts 1 or 2), whereas the pests+viruses ranged from 18% to 33% host genus specialists (median 4 or 5) (Fig. 2, Table S3).

The median plant genus had 52 recorded pests (minimum 7, maximum 426, of a total of 1670 pest species in the database). The median number of pests per host genus was 32 for insects, 7 for fungi, 4 for mites, and 1 pest per host genus for the remaining pest groups (Fig. S2, Table S5).

### Phylogenetic signal in host ranges of different kinds of plant pests

**Figure 3:**
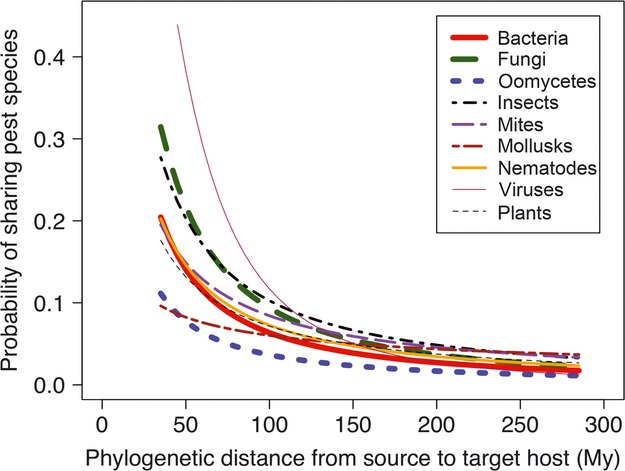
Phylogenetic signal in the likelihood that plants share a common pest. Phylogenetic signal in the probability that a pest or pathogen from a source host also attacks a target host. Curves are predicted from logistic regressions for each major group of pests, using coefficients as given in Table 1. All slopes except mollusks are significantly different from zero. Additional details in Figs S3-S5.

The probability that two host genera share a pest or pathogen declined significantly with phylogenetic distance between the hosts, for all groups of pest and pathogens except mollusks (Table 1, Figs 3 and S3–S5). Viruses show a significantly steeper slope than other groups, but the remaining groups show significant overlap in estimates of the slope of the phylogenetic signal (Table 1, Fig. S3). The directly comparable logistic regression from empirical host range testing of necrotrophic fungal pathogens of tropical tree leaves (Gilbert and Webb [Bibr b20]) was logit(S) = 2.9113 − 1.5944*[log_10_(distance + 1)]. Estimates for a much more phylogenetically diverse collection of fungi from the GPDD showed a similar pattern, but with a steeper slope for the effect of phylogenetic distance (median = −3.3249; 95% CI −3.7439 to −2.8019).

**Table 1:** Phylogenetic signal in pest sharing between plant host genera

	Coefficients		95% confidence intervals	
		
Pest group (*n*)	β_0_	β_1_	β_0_	β_1_
Bacteria (30)	3.2613	−2.9706	1.5581–4.3791	−3.4605 to −2.2215
Fungi (95)	4.3961	−3.3249	3.1958–5.3539	−3.7439 to −2.8019
Oomycetes (32)	2.0763	−2.6679	−0.8020–3.6867	−3.3745 to −1.4216
Insects (637)	3.2441	−2.7004	2.7108–3.7216	−2.9073 to −2.4694
Mites (87)	1.9584	−2.1681	0.3266–2.9620	−2.6039 to −1.4625
Mollusks (37)	−0.4667	−1.1391	−4.5587–1.5261	−1.9896 to –0.5920
Nematodes (70)	2.7157	−2.6249	1.8376–3.4603	−2.9498 to −2.2445
Viruses (108)	8.4044	−5.2014	7.7856–8.9129	−5.4320 to −4.9236
Plants (53)	1.9979	−2.2775	1.0617–2.5609	−2.5233 to −1.8644

Shown are the coefficients of logistic regressions (median and 95% confidence intervals) of whether target host genus was known to be susceptible (S) to a pest from a source host genus, as a function of the phylogenetic distance between source and target hosts. The regression takes the form of logit(S) = β_0_ + β_1_^*^log_10_(PD + 1), where PD is the phylogenetic distance (time of independent evolution in My) between the source and target host genera. All pest groups except mollusks had phylogenetic signals significantly different from zero (95%CI for β_1_ did not overlap 0). The probability that a target host is susceptible to a pest from a source host is then prob(susceptible) = exp[logit(S)]/[1 + exp(logit(S))]. Number of pest species included in the regression (those with >1 known host genus) is given in parentheses as *n*.

### Interaction between host breadth and phylogenetic signal

**Figure 4:**
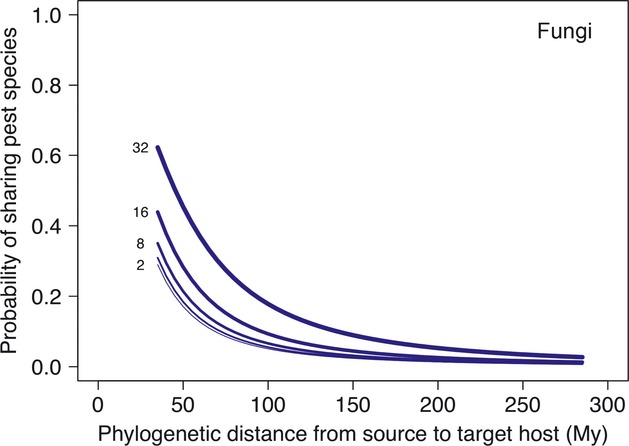
Known host breadth of pests affects the likelihood that plants will share a pest. Effect of known number of host genera attacked by a fungal pathogen on the phylogenetic signal in the probability that a pest or pathogen from a source host also attacks a target host. Curves are predicted from the logistic regression with coefficients given in Table 2 for phylogenetic distance between hosts and the number of hosts known for a particular pathogen (shown as numbers to the left of each curve). Curves for all nine groups of pest and pathogens take a similar form and are shown in Fig. S6.

We expected that pests with a greater number of known hosts would be more likely to share multiple hosts across all phylogenetic distances (i.e., the probability curves would shift upward with more known hosts). Analyses of each of the nine groups of pests and pathogens supported this expectation (Figs 4 and S6). The coefficients for phylogenetic distance were all significantly lower than zero, and coefficients for the number of known hosts were all significantly greater than zero (Table 2). We included only the main effects in the model, because when we included the interaction term in the model (i.e., Phylogenetic distance*Number known hosts), the interaction term was significantly different from zero for only two groups, and then, the main effect of known hosts was not significant (Table S6).

**Table 2:** Effect of host breadth and phylogenetic distance in the likelihood the two hosts share a pest

	Coefficients			95% confidence intervals		
		
Pest group	β_0_	β_1_	β_2_	β_0_	β_1_	β_2_
Bacteria	3.7354	−3.5794	0.0664	1.7502–5.2889	−4.2790 to −2.7080	0.0634–0.0711
Fungi	6.0593	−4.5293	0.0466	5.0467–6.7900	−4.8649 to −4.0673	0.0455–0.0483
Oomycetes	3.2131	−3.5290	0.0818	0.3439–4.9863	−4.3281 to −2.2792	0.0788–0.0918
Insects	3.4837	−3.2188	0.0532	2.9285–3.9645	−3.4273 to −2.9736	0.0526–0.0539
Mites	2.6463	−2.8747	0.0447	1.1852–3.6436	−3.3190 to −2.2357	0.0438–0.0462
Mollusks	−1.2019	−1.1576	0.0474	−3.7591–0.8348	−2.0378 to −0.0713	0.0467–0.0495
Nematodes	2.2984	−2.9162	0.0884	1.3244–3.1807	−3.3146 to −2.4921	0.0857–0.0932
Viruses	8.3535	−5.6544	0.0967	7.5721–9.0395	−5.9872 to −5.2985	0.0904–0.1029
Plants	2.2164	−2.7873	0.0727	1.2404–2.8660	−3.0934 to −2.3506	0.0704–0.0782

Given are the coefficients of logistic regressions (median and 95% confidence intervals) of phylogenetic signal in host sharing with two independent variables: phylogenetic distance between source host genus and target host genus (coefficient β_1_) and the number of known hosts for the pest (coefficient β_2_). The dependent variable was whether the target host genus was known to be susceptible (S) to a pest from the source host genus. The regression takes the form of logit(S) = β_0_ + β_1_^*^log_10_(PD + 1) + β_2_^*^(Number of known hosts), where PD is the phylogenetic distance (time of independent evolution in My) between the source and target host genera. Note that interaction term was not significant (S6), so the models presented here were run with main effects only. The number of pests in each group is the same as in Table 1. Coefficient β_1_ was significantly negative and β_2_ was significantly positive for all nine pest and pathogen groups (95% CI did not overlap zero).

## Discussion

### Structure of host ranges and pest diversity in the GPDD

The number of hosts known to be attacked by a particular pest or pathogen and the number of pests and pathogens known from a particular plant genus are both likely to underestimate the true numbers. Exhaustive surveys of host range or pest associates are rare and then are usually restricted to small geographic areas. Nevertheless, the global scope of the GPDD data provides the best current opportunity to examine the structure of plant–pest associations for a broad diversity of plants and pests of economic and ecological importance.

Microbial pathogens (fungi, bacteria, and oomycetes) showed significantly more host specialization than did the pests (insects, mites, mollusks, nematodes, parasitic plants) and viruses. The structure of host ranges for viruses may be more similar to that of pests than pathogens because the host range of many plant viruses is functionally determined by that of their arthropod vectors (Gray and Banerjee 1999). The shape of the histograms (Fig. S1), as well as the ‘S’ shape of the cumulative distribution curves for the pest + virus groups (Fig. 2), suggests the possibility of superimposed curves from two distinct life-history strategies: one strongly host specialized and another more polyphagous, the second with a peak of host diversity at 4–15 plant genera. Feeding guilds of tropical herbivorous insects show strikingly different patterns of host specificity, ranging from near monophagy to extreme generalization (Novotny et al. 2010). Similarly, obligately biotrophic pathogenic fungi such as rusts and smuts tend to have much narrower host ranges than do facultative necrotrophic pathogens (Oliver and Ipcho 2004). Careful additional study is merited on how life-history strategies affect the breadth and phylogenetic signal in host range for plant pests and pathogens. Finer subdivisions of pests than the nine groups used here may provide even more precision in evaluating expected host ranges of plant pests and pathogens.

### Phylogenetic signal in host ranges of different kinds of plant pests

All groups of pests and pathogens showed strong phylogenetic signals in their host ranges (Fig. 3, Table 1, Figs S3–S5), with the exception of mollusks. The estimates of slopes of phylogenetic signal are conservative, because the regressions exclude intrageneric comparisons (i.e., any pests and pathogens that are limited to a single host genus), because their inclusion would force the intercept through 100% probability of sharing. Even so, the intercepts for all the pest groups with significant slopes (excluding mollusks) correspond to a range of 88% to 99% (median 95%) likelihood of sharing at a phylogenetic distance that approaches zero (Fig. S5). Estimates of the intercept values should be treated with some caution, because they represent an extrapolation beyond the range of data used to parameterize the models.

The slopes for phylogenetic signal presented here were usually steeper than those found in empirical host range testing of necrotrophic fungal pathogens of tropical tree leaves (for all pest groups except oomycetes, mites, and mollusks) (Gilbert and Webb [Bibr b20]). There are several reasons to expect steeper slopes from analysis of the GPDD data than from the limited empirical data from a tropical rainforest. First, Gilbert and Webb ([Bibr b20]) limited their analysis to necrotrophic fungal pathogens that were easily grown on laboratory media, which might be expected to have less specific host requirements than many of the fungi (and other pests) in the current analysis. Second, fungi and plants used in the empirical tests all co-occurred in a small area (0.79 ha), where fungi and local taxa should have had abundant ecological opportunity for evolutionary acquisition of a range of hosts (even distantly related) through frequent local encounters. The third reason, however, may be an artifact of sampling effort. The host range testing by Gilbert and Webb ([Bibr b20]) was intentionally phylogenetically broad, testing for pathogenicity on hosts ranging across the full spectrum of phylogenetic distances among angiosperms in a tropical forest. This would make it likely to uncover occasional hosts at large phylogenetic distances from the source host. In contrast, many local host range testing reports focus on close relatives from a local region (Wapshere 1974; Briese 2003; Barton 2004). This is a reasonable approach when resources are limited because those are the plants that are mostly likely to be hosts and to have an agronomic or ecological impact, but a lack of testing of distantly related plants could mean that the GPDD data would be somewhat more likely to have false zeros at large phylogenetic distances. On the other hand, this may be offset by field reports that inform the GPDD of emerging diseases across broad geographic areas that would likely include a broad phylogenetic sample. Extensive, carefully structured host range testing will be necessary to determine the relative importance of sampling bias, life-history variation, and evolutionary opportunity in shaping the steepness of the phylogenetic signal in host range.

### Host breadth and phylogenetic signal

The combination of phylogenetic distance between hosts and the number of known hosts of a pathogen provided clearer predictions of the likelihood that two hosts would share a pest or pathogen. A pest or pathogen with a large number of hosts reported in the GPDD is likely to truly have a broad host range, including a large number of as yet undescribed hosts. In nearly any local assemblage of potential plant hosts, there will be few pairs of closely related host genera, and many more combinations of genera that are more distantly related. This means that when a pest has more than a small number of hosts, each additional host would necessarily also expand the phylogenetic breadth of the host range, even when there is a phylogenetic signal in host range (Fig. 1).

### Applications

The *Pest Risk Analysis for Quarantine Pests* [International Plant Protection Convention (IPPC) ISPM No. 11] was established in 2001 (amended in 2004) as part of the IPPC International Standards for Phytosanitary Measures (Secretariat of the International Plant Protection Convention 2004). The IPPC has 177 government signatories and provides the standards for phytosanitary policies and actions of USDA APHIS-PPQ and its national and regional equivalents around the world [e.g., the European Plant Protection Organization (EPPO) and the meso-American Organismo International Regional de Sanidad Agropecuaria (OIRSA)]. Along with suitable environmental conditions and agricultural practices, the IPPC ISPM 11 standards focus on the importance of the presence, abundance, and distribution of suitable host species for an introduced plant pest to establish and spread in a new area. Compiled lists of known hosts of plant pests and pathogens are the current standard for assessing which hosts in the new area are most likely threatened. The analytical tools presented here enhance the power of such databases by allowing interpolation and extrapolation to novel host–pest combinations.

Our results stress that for a broad diversity of plant pests and pathogens, imposing a step-function decision process (e.g., all hosts within a genus are at risk, and others are not) is not ideal for risk analysis for novel pests and pathogens. Instead, the probability that plant species will share pests declines as a continuous function of phylogenetic distance between the plant species. Each known host of a pest can be used as the source host for calculating phylogenetic distances to target hosts and then to predict which other hosts are likely targets. The combined predictions can be combined to estimate the probability of sharing or create rank-order lists of at-risk plant taxa.

The number of known hosts of a pest or pathogen is a good indicator of the expected overall breadth of hosts for a pest or pathogen, which, when combined with the phylogenetic signal in host range, presents a simple predictor of *which* hosts a particular pest or pathogen is likely to attack. The logistic equations developed here for each of nine major groups of plant pests and pathogens require knowing (i) the major evolutionary clade to which the pest belongs (e.g., fungi, mites), (ii) at least one genus of known hosts for the pathogen or pest, and (iii) the genera of plants of concern (i.e., regionally important wildland species and agricultural crops). Predictions from each of the known host genera could be combined to provide more complete predictions of expected hosts. Even for novel pests with very limited knowledge of host range, the models provide significantly more information on which to base pest risk analyses than the current use of host range lists.

It is important to note that this model is useful to predict which hosts are likely to be susceptible to a particular pest or pathogen, but does not indicate whether the impact of those pests on a given host will be severe or benign. That is, the models are designed to provide information on likely incidence, but not about severity of damage or on the broader economic or environmental effects of an introduced pest. The impact on individual hosts is sometimes greater for pests and pathogens with more specialized host ranges (Agudelo-Romero and Elena 2008), whereas the impacts on ecological systems may be differ depending on whether the pests are more specialized or have broader host ranges (Shearer et al. 2007; Dyer et al. 2010). Whether phylogenetic tools can be used to evaluate relative impacts on individual hosts and ecological systems are important next steps.

The movement of live plants for horticultural use is a particularly effective and common pathway for pest introductions (Campbell 2001). The host-to-host based phylogenetic approach we present here offers an integrated index of the likelihood that unknown pests and pathogens that hitch-hike on stock of particular horticulture species would pose a threat to the environmentally and economically important plant species in a proposed area of introduction. Insights from phylogenetic ecology of host ranges thus provide a new tool for evaluating the risk of plant introductions in the absence of exhaustive knowledge of the pests of introduced plant species.

## Data archiving statement

The Global Pest and Disease Database is an expert-user database created and maintained by the USDA Animal and Plant Health Inspection Service – Plant Protection and Quarantine Division (APHIS-PPQ). Because of the politically sensitive nature of phytosanitary or trade regulations based in part on these databases, USDA makes the data available only to researchers through contractual cooperative agreements. Contact K. Suiter for further information. A redacted version of the data used for these analyses, with pest names replaced with codes, is available as a comma-delimited matrix in Data S1.

## Supporting Information

**Data S1.** Comma-delimited matrix of data from Global Pest and Disease Database used in this study.

**Figure S1.** Number of pest species with number of known hosts, separately for each major group of pests.

**Figure S2.** Cumulative proportion of all 210 host genera with increasing numbers of pest species reported from a given host genus.

**Figure S3.** Comparison of slopes of phylogenetic signal of host sharing from the logistic regressions.

**Figure S4.** Predicted sharing of hosts from logistic regressions of phylogenetic signal of host sharing, extended to 0-My phylogenetic distance.

**Figure S5.** Logistic regressions for phylogenetic signal of host sharing for each major group of pathogen or pest.

**Figure S6.** Logistic regressions of host sharing with phylogenetic distance and number of known hosts as the independent variables.

**Table S1.** Genera of angiosperm plant hosts selected from the APHISPPQ Global Pest and Disease Database for this study.

**Table S2.** Genera of known pests of the 210 genera of Angiosperms selected from the APHIS-PPQ Global Pest and Disease Database.

**Table S3.** Kolmogorov–Smirnov tests to compare empirical cumulative distribution functions for number of known hosts for each major group of pests.

**Table S4.** Descriptive moments of the number of known hosts for each major group of pests.

**Table S5.** Descriptive moments of the number of known pests from each major group on the 210 host genera.

**Table S6.** Coefficients of logistic regressions of phylogenetic distance, number of known hosts, and the interaction, on host sharing.
