# Supplementary material for: Evolutionary tools for phytosanitary risk analysis: phylogenetic signal as a predictor of host range of plant pests and pathogens
Source: Evol Appl. 2012 May 3;5(8):869–78. doi: 10.1111/j.1752-4571.2012.00265.x (PMC3552404; doi:10.1111/j.1752-4571.2012.00265.x)
Supplement: Supplementary file 2 [file eva0005-0869-SD2.docx]

**Dataset S1.** Comma-delimited matrix of data from Global Pest and Disease Database used in this study. The matrix includes angiosperm host genera recorded for each pest species (1 indicates that species within that plant genus are known as hosts). Pest names are redacted and replace by names that indicate the major pest group and a code number. The Global Pest and Disease Database is an expert-user database created and maintained by the USDA Animal and Plant Health Inspection Service – Plant Protection and Quarantine Division (APHIS-PPQ).  Because of the politically sensitive nature of phytosanitary or trade regulations based in part on these databases, USDA makes the complete pest data available only to researchers through contractual cooperative agreements.  Contact K. Suiter for further information.
