# Supplementary material for: Evolutionary tools for phytosanitary risk analysis: phylogenetic signal as a predictor of host range of plant pests and pathogens
Source: Evol Appl. 2012 May 3;5(8):869–78. doi: 10.1111/j.1752-4571.2012.00265.x (PMC3552404; doi:10.1111/j.1752-4571.2012.00265.x)
Supplement: Supplementary file 3 [file eva0005-0869-SD3.pdf]

Supporting information for

Evolutionary Tools for Phytosanitary Risk Analysis: Phylogenetic Signal as a Predictor of Host Range of Plant Pests and Pathogens

FIGURES

Figure S1. Number of pest species with number of known hosts, separately for each major group of pests.

Figure S2. Cumulative proportion of all 210 host genera with increasing numbers of pest species reported from a given host genus.

Figure S3. Comparison of slopes of phylogenetic signal of host sharing from the logistic regressions.

Figure S4. Predicted sharing of hosts from logistic regressions of phylogenetic signal of host sharing, extended to 0-My phylogenetic distance.

Figure S5. Logistic regressions for phylogenetic signal of host sharing for each major group of pathogen or pest.

Figure S6. Logistic regressions of host sharing with phylogenetic distance and number of known hosts as the independent variables.

TABLES

Table S1. Genera of angiosperm plant hosts selected from the APHIS-PPQ Global Pest and Disease Database for this study.

Table S2. Genera of pests of the 210 genera of Angiosperms selected from the APHIS-PPQ Global Pest and Disease Database.

Table S3. Kolmogorov-Smirnov Tests to compare empirical cumulative distribution functions for number of known hosts for each major group of pests.

Table S4. Descriptive moments of the number of known hosts for each major group of pests.

Table S5. Descriptive moments of the number of known pests from each major group on the 210 host genera.

Table S6. Coefficients of logistic regressions of phylogenetic distance, number of known hosts, and the interaction, on host sharing.

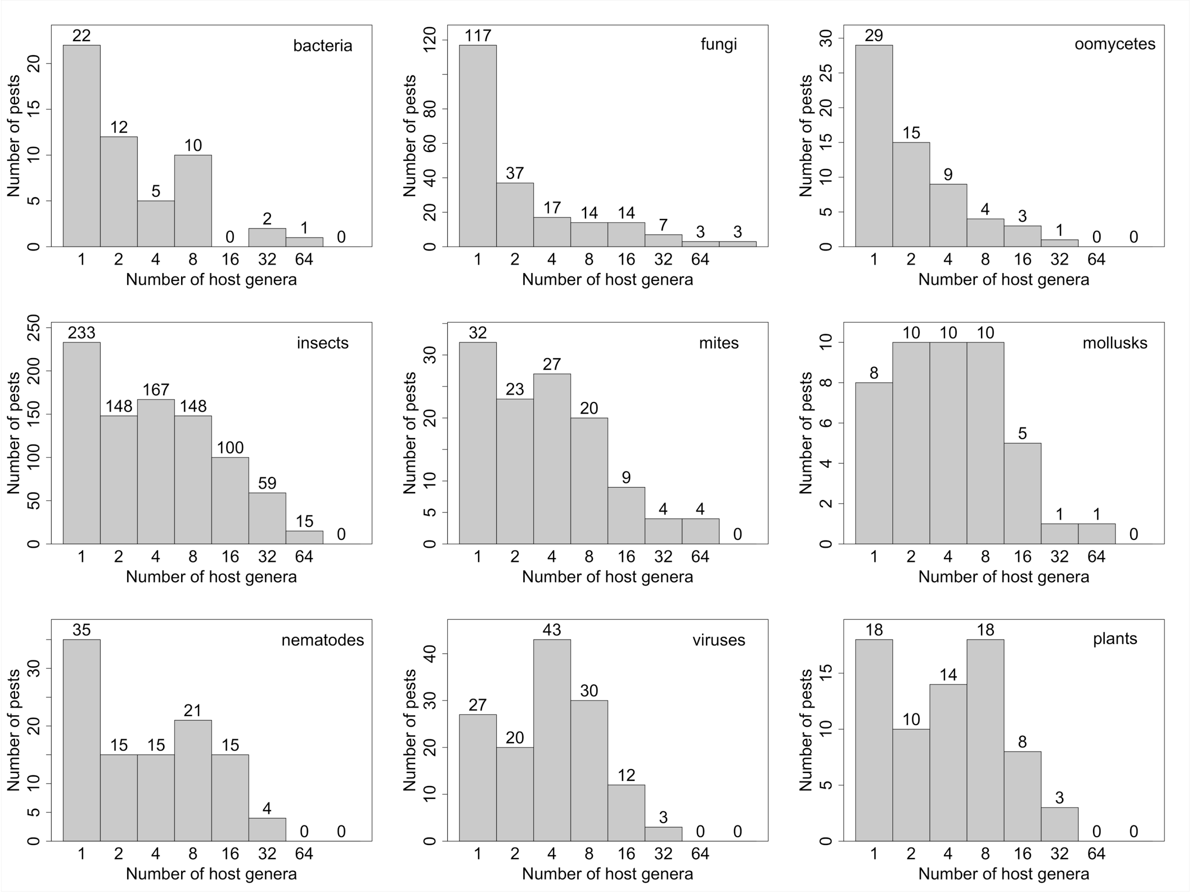

Figure S1. Number of pest species with number of known hosts, separately for each major group of pests. Number of host genera are grouped in Preston octave log2(host number) series (i.e., 1, 2-3, 4-7, 8-15, 16-31,32-63, 64-127, 128+). Pest species included 52 bacteria, 212 fungi, 61 oomycetes, 870 insects, 119 mites, 45 mollusks, 105 nematodes, 71 parasitic plants, and 135 viruses. These data correspond to those in Figure 2 in the main text.

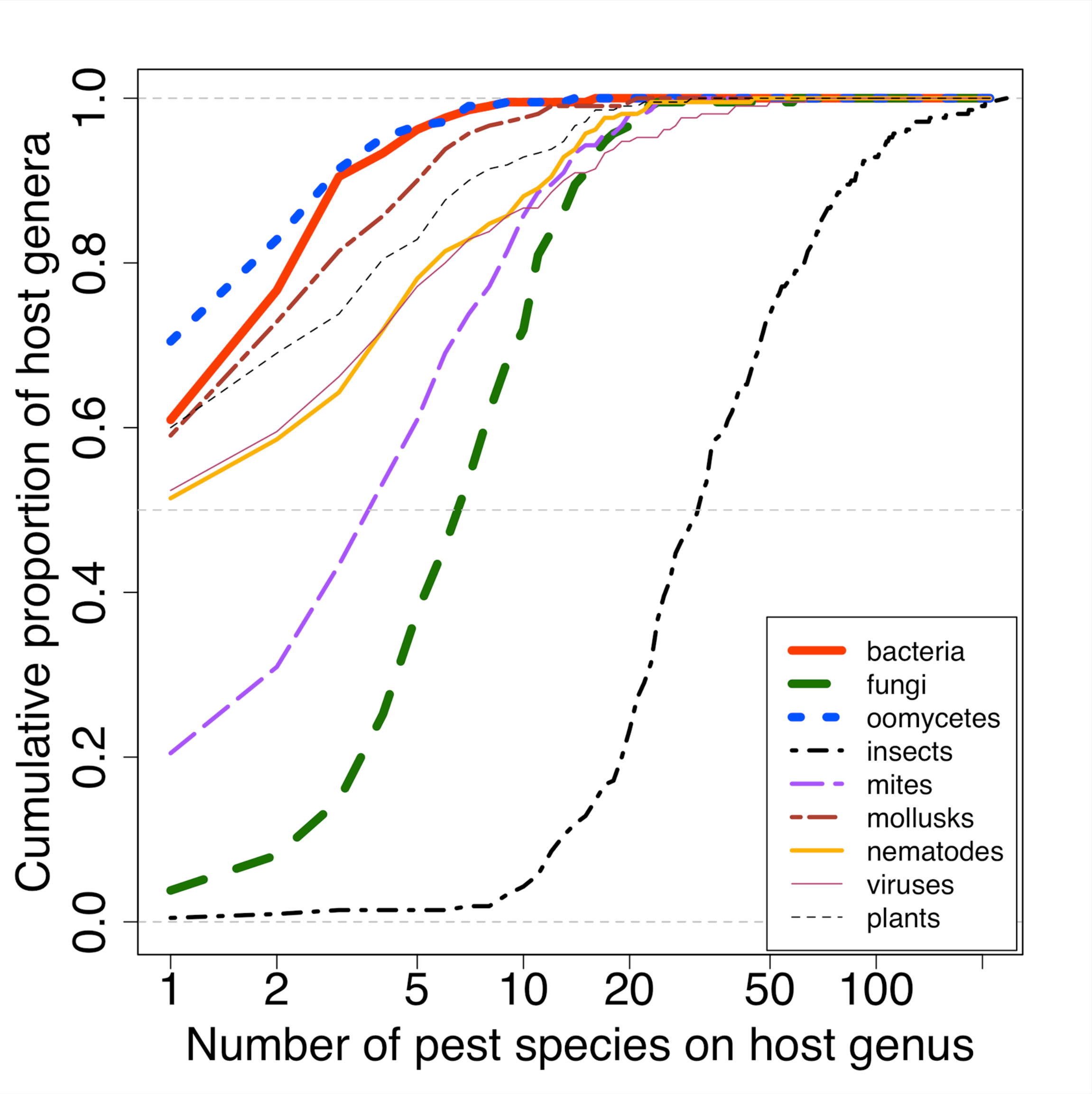

Figure S2. Cumulative proportion of all 210 host genera with increasing numbers of pest species reported from a given host genus.

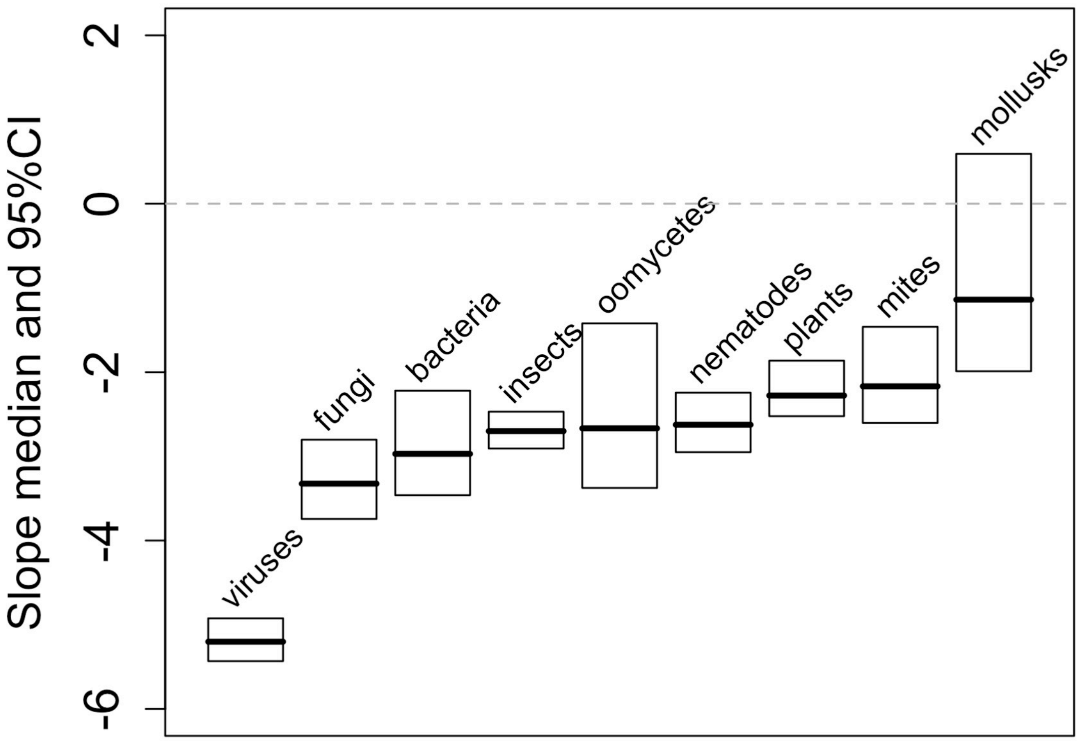

Figure S3. Comparison of slopes of phylogenetic signal of host sharing from the logistic regressions. Medians and 95% confidence intervals are as given in Table 1 in the text. The regression takes the form of logit(susceptible)=Intercept + Slope * $\log_{10}$(PD +1), where PD is the phylogenetic distance (time of independent evolution in My) between the source and target hosts. The probability that a target host is susceptible to a pest from a source host is then prob(susceptible) =exp(logit(susceptible)) / [1 + exp(logit(susceptible))]. Shown are the median slopes (thick horizontal lines) and 95% confidence intervals (top and bottom of boxes) for the slopes. All slopes except for mollusks are significantly different from zero (95% CI does not include zero). Viruses show a significantly steeper slope than other groups, but the remaining groups show significant overlap in estimates of the slope of the phylogenetic signal.

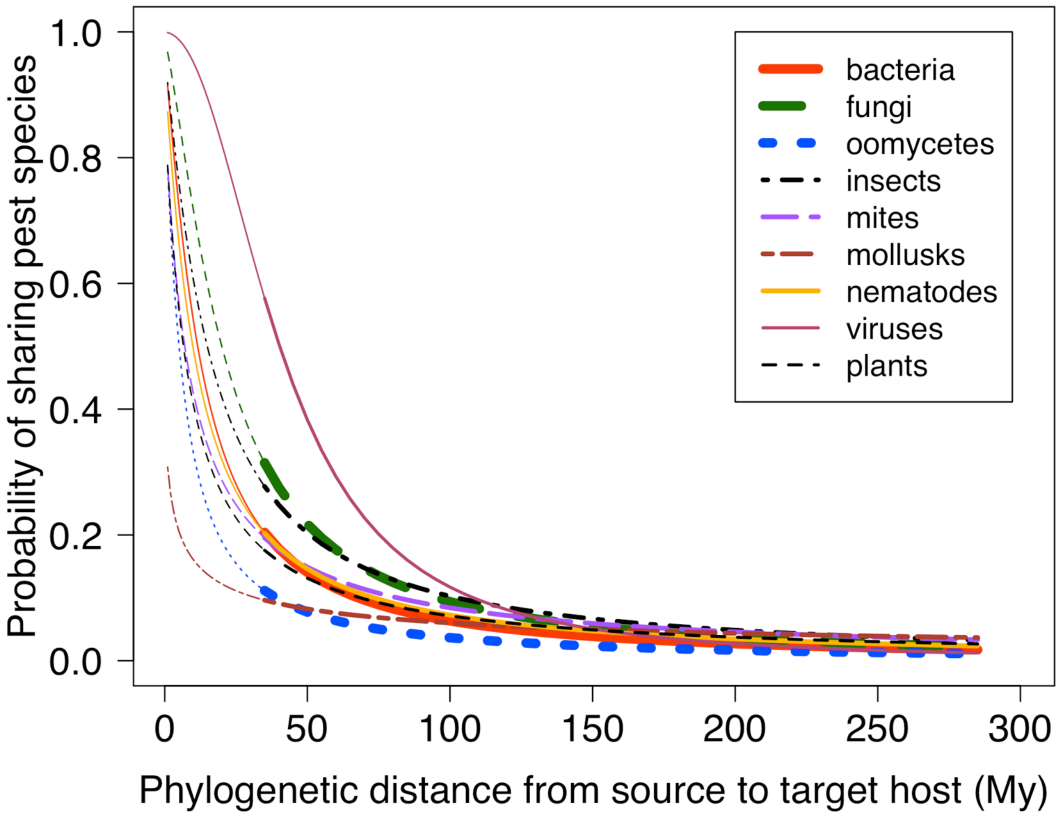

Figure S4. Predicted sharing of hosts from logistic regressions of phylogenetic signal of host sharing, extended to 0-My phylogenetic distance. Curves use the same formulas as given in Fig. 2 and Table 1 in the text. Fine lines from 0 to 35 My phylogenetic distance extend the predicted probability of sharing beyond the range of phylogenetic distances used in parameterizing the model.

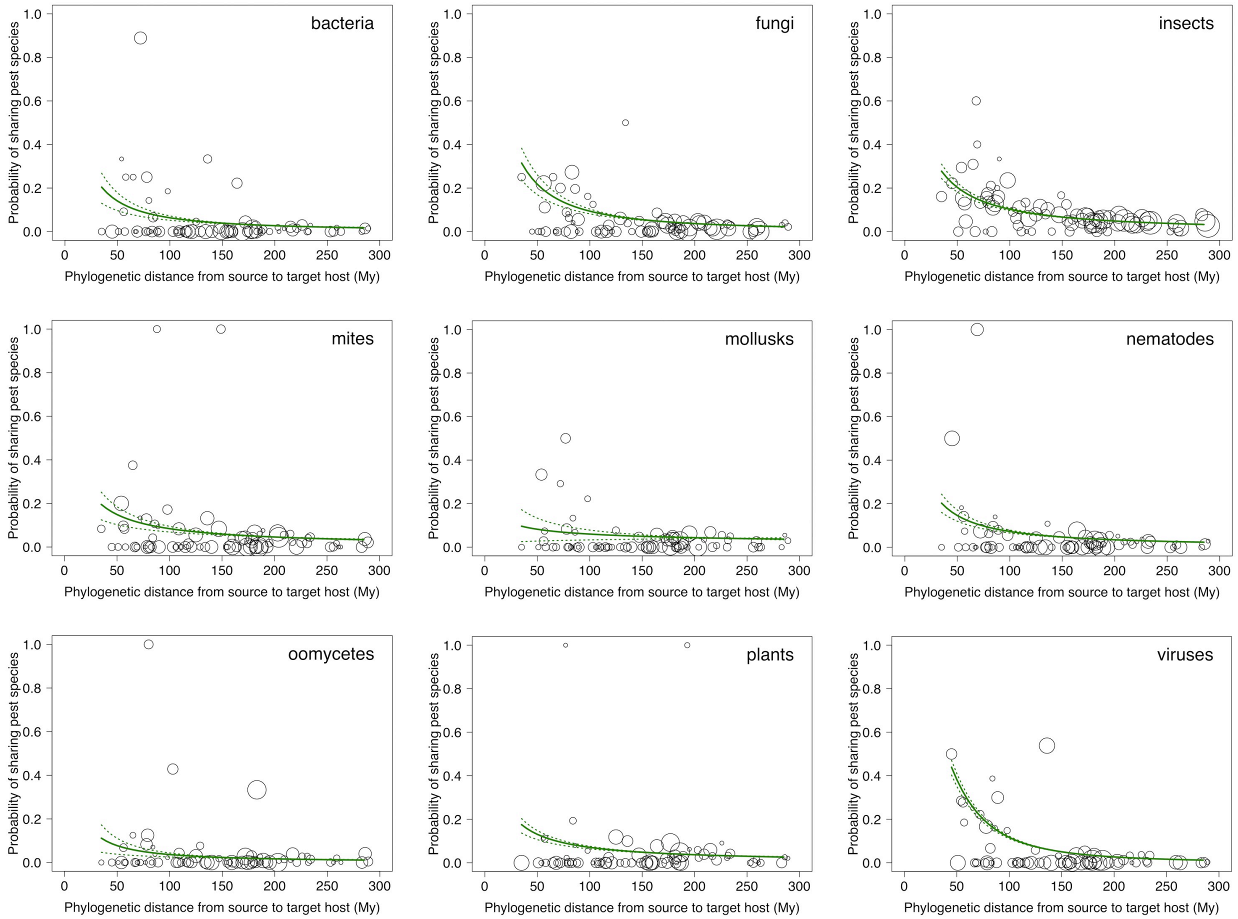

Figure S5. Logistic regressions for phylogenetic signal of host sharing for each major group of pathogen or pest. Circles indicated the median proportion of source-target pairs at that phylogenetic distance (in 1-My intervals) that were susceptible. The size of the circle is proportional to the number of plant pairs at that distance.

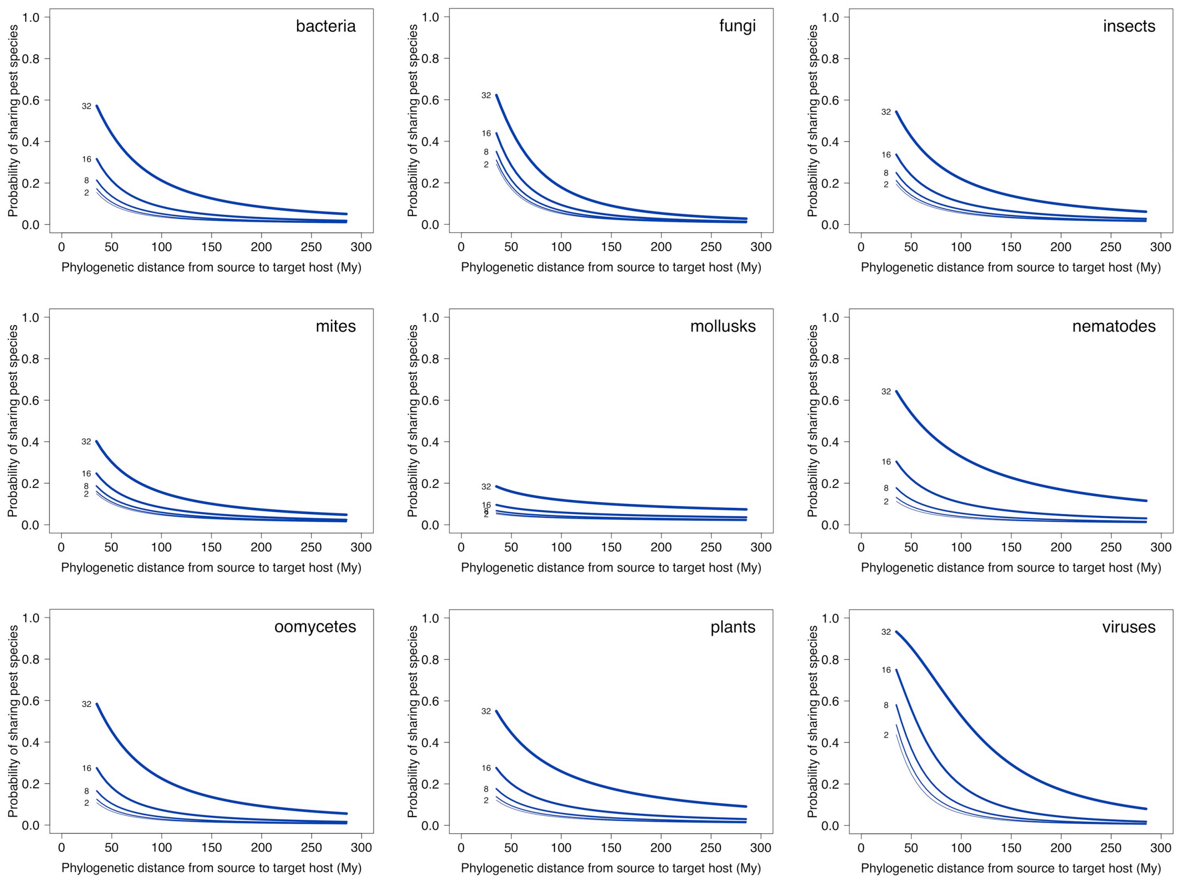

Figure S6. Logistic regressions of host sharing with phylogenetic distance and number of known hosts as the independent variables. The probability that a pest or pathogen from one host genus (source) will also be found on another host genus (target) can be calculated using the phylogenetic distance between the source and target host genera) and the number of known hosts of the pest or pathogen, and parameterized using the coefficients given in Table 2, as follows: $\text{logit}(S) = \beta_0 + \beta_1 * \log_{10}(PD + 1) + \beta_2 * (\text{Number of known hosts})$, where prob(target in susceptible) = exp(logit(S)) / [1 + exp(logit(S))]

Table S1. Genera of angiosperm plant hosts selected from the APHIS-PPQ Global Pest and Disease Database for this study.

Abelmoschus	*Acacia*	*Acalypha*	*Acer*	*Actinidia*	*Aesculus*	*Agropyron*
Albizia	*Allium*	*Alnus*	*Amaranthus*	*Anacardium*	*Ananas*	*Annona*
Anthurium	*Apium*	*Arachis*	*Areca*	*Artemisia*	*Artocarpus*	*Asparagus*
Avena	*Averrhoa*	*Bambusa*	*Bauhinia*	*Berberis*	*Beta*	*Betula*
Brassica	*Bromus*	*Cajanus*	*Calophyllum*	*Camellia*	*Capsicum*	*Carica*
Carya	*Cassia*	*Castanea*	*Casuarina*	*Chenopodium*	*Chrysanthemum*	*Chrysophyllum*
Cicer	*Cichorium*	*Cinnamomum*	*Citrullus*	*Citrus*	*Clerodendrum*	*Cocos*
Codiaeum	*Coffea*	*Colocasia*	*Convolvulus*	*Corchorus*	*Corylus*	*Crataegus*
Crotalaria	*Croton*	*Cucumis*	*Cucurbita*	*Cydonia*	*Cynara*	*Cynodon*
Cyperus	*Dahlia*	*Datura*	*Daucus*	*Dianthus*	*Digitaria*	*Dimocarpus*
Dioscorea	*Diospyros*	*Dracaena*	*Echinochloa*	*Elaeis*	*Eleusine*	*Elymus*
Eriobotrya	*Erythrina*	*Eucalyptus*	*Eugenia*	*Euonymus*	*Euphorbia*	*Fagus*
Festuca	*Ficus*	*Fortunella*	*Fragaria*	*Fraxinus*	*Garcinia*	*Gardenia*
Gladiolus	*Glycine*	*Gossypium*	*Hedera*	*Helianthus*	*Hemerocallis*	*Hevea*
Hibiscus	*Hordeum*	*Humulus*	*Ilex*	*Ipomoea*	*Jasminum*	*Juglans*
Lactuca	*Lagerstroemia*	*Lantana*	*Lathyrus*	*Lens*	*Leucaena*	*Ligustrum*
Linum	*Litchi*	*Lolium*	*Lonicera*	*Lotus*	*Luffa*	*Lupinus*
Lycopersicon	*Macadamia*	*Magnolia*	*Malus*	*Mangifera*	*Manihot*	*Manilkara*
Medicago	*Melia*	*Mentha*	*Momordica*	*Morus*	*Murraya*	*Musa*
Nephelium	*Nerium*	*Nicotiana*	*Ocimum*	*Olea*	*Opuntia*	*Oryza*
Panicum	*Paspalum*	*Passiflora*	*Pelargonium*	*Pennisetum*	*Persea*	*Petunia*
Phaseolus	*Phoenix*	*Physalis*	*Pieris*	*Piper*	*Pistacia*	*Pisum*
Pittosporum	*Plantago*	*Platanus*	*Plumeria*	*Poa*	*Polygonum*	*Poncirus*
Populus	*Pouteria*	*Prunus*	*Psidium*	*Pueraria*	*Punica*	*Pyrus*
Quercus	*Raphanus*	*Rhamnus*	*Rhododendron*	*Rhus*	*Ribes*	*Ricinus*
Robinia	*Rosa*	*Rubus*	*Rumex*	*Saccharum*	*Salix*	*Salvia*
Secale	*Senecio*	*Setaria*	*Sida*	*Solanum*	*Sonchus*	*Sorbus*
Sorghum	*Spinacia*	*Spondias*	*Syringa*	*Syzygium*	*Tectona*	*Terminalia*
Theobroma	*Tilia*	*Trifolium*	*Triticum*	*Ulmus*	*Urtica*	*Vaccinium*
Viburnum	*Vicia*	*Vigna*	*Vitis*	*Zea*	*Zinnia*	*Ziziphus*

Table S2. Genera of pests of the 210 genera of Angiosperms selected from the APHIS-PPQ Global Pest and Disease Database. A total of 1670 pest species, including 52 bacteria, 215 fungi, 58 oomycetes, 875 insects, 114 mites, 45 mollusks, 105 nematodes, 71 parasitic plants, and 135 viruses were used in this analysis.

**BACTERIA**						
Burkholderia	*Candidatus*	*Clavibacter*	*Erwinia*	*Pantoea*	*Phytoplasma*	*Pseudomonas*
Ralstonia	*Rathayibacter*	*Xanthomonas*	*Xylella*	*Xylophilus*		
**FUNGI**						
Acremonium	*Actinodochium*	*Aecidium*	*Alternaria*	*Apiognomonia*	*Armillaria*	*Ascochyta*
Aspergillus	*Asterina*	*Asterolibertia*	*Athelia*	*Beltraniopsis*	*Bipolaris*	*Botryosphaeria*
Botryotinia	*Calonectria*	*Candida*	*Capnodium*	*Catacauma*	*Caudella*	*Ceratobasidium*
Ceratocystis	*Cercospora*	*Chaetothyrina*	*Chaetothyrium*	*Chalara*	*Chrysomyxa*	*Ciliochorella*
Claviceps	*Cochliobolus*	*Coleosporium*	*Colletotrichum*	*Coniella*	*Curvularia*	*Cytosphaera*
Diaporthe	*Dichotomophthora*	*Didymella*	*Diplodia*	*Dolabra*	*Elsinoe*	*Entyloma*
Ephelis	*Erysiphe*	*Erythricium*	*Fulvia*	*Fusarium*	*Gibberella*	*Glomerella*
Guignardia	*Gymnopus*	*Gymnosporangium*	*Harpophora*	*Helicobasidium*	*Hemileia*	*Hendersonia*
Lachnellula	*Leveillula*	*Macrophoma*	*Macrophomina*	*Magnaporthe*	*Mauginiella*	*Melampsora*
Melanomma	*Microcyclus*	*Microsphaera*	*Monilinia*	*Moniliophthora*	*Mycena*	*Mycosphaerella*
Mycovellosiella	*Myrothecium*	*Myxosporium*	*Nectria*	*Oidiopsis*	*Oncobasidium*	*Oospora*
Ophiostoma	*Parkerella*	*Penicillium*	*Pestalotia*	*Pestalotiopsis*	*Phacidiopycnis*	*Phaeoramularia*
Phaeosphaeria	*Phakopsora*	*Phellinus*	*Phialophora*	*Phlyctema*	*Phoma*	*Phomopsis*
Phragmidium	*Phyllosticta*	*Physoderma*	*Polyscytalum*	*Pseudocercospora*	*Pseudocercosporella*	*Pseudocochliobolus*
Pseudopezicula	*Puccinia*	*Pyricularia*	*Raffaelea*	*Ramulispora*	*Rhacodiella*	*Rhizoctonia*
Rhizopus	*Rhizopycnis*	*Rosellinia*	*Sawadaea*	*Sclerotinia*	*Septoria*	*Setosphaeria*
Sphaceloma	*Stemphylium*	*Stigmina*	*Synchytrium*	*Thanatephorus*	*Thecaphora*	*Thekopsora*
Tilletia	*Uredo*	*Urocystis*	*Uromyces*	*Uromycladium*	*Venturia*	*Verticillium*
Waitea						
**OOMYCETES**						
Peronosclerospora	*Phytophthora*	*Pythium*	*Sclerophthora*	*Trachysphaera*		
**INSECTS**						
Abgrallaspis	*Aburina*	*Acalolepta*	*Acalymma*	*Acanthocoris*	*Acanthophorus*	*Acanthoscelides*
Acanthosoma	*Accuminulia*	*Achaea*	*Acherontia*	*Acia*	*Acizzia*	*Acledra*
Acrida	*Acrobasis*	*Acrocercops*	*Acrolepiopsis*	*Acromyrmex*	*Acronicta*	*Acutaspis*
Acyrthosiphon	*Adelphocoris*	*Adoretus*	*Adoxophyes*	*Aedia*	*Aegoceropsis*	*Aegorhinus*
Aelia	*Aeolesthes*	*Aeolothrips*	*Aetalion*	*Aetheomorpha*	*Aethina*	*Aglais*
Aglaope	*Agrilus*	*Agrius*	*Agrotis*	*Akermes*	*Alcidodes*	*Aleurocanthus*
Aleurocerus	*Aleuroclava*	*Aleurodicus*	*Aleurolobus*	*Aleuroplatus*	*Aleurothrixus*	*Aleurotrachelus*
Aleurotuberculatus	*Aleyrodes*	*Alkindus*	*Aloa*	*Aloephagus*	*Alpenus*	*Altica*

Amasa	*Amauromyza*	*Amblypelta*	*Amblyrrhinus*	*Ambrosiodmus*	*Amphipyra*	*Amphorophora*
Amradiplosis	*Amrasca*	*Amritodus*	*Amsacta*	*Anacanthocoris*	*Anacridium*	*Anarsia*
Anascirtothrips	*Anastrepha*	*Ancognatha*	*Andaspis*	*Anomala*	*Anomis*	*Anoplocnemis*
Anoplolepis	*Anoplophora*	*Antestiopsis*	*Anthene*	*Anthonomus*	*Anticarsia*	*Aonidiella*
Apate	*Aphanisticus*	*Aphanostigma*	*Aphis*	*Aphrophora*	*Apis*	*Aploneura*
Apoderus	*Apogonia*	*Apolygus*	*Aporia*	*Apriona*	*Apsylla*	*Arboridia*
Archips	*Archipsocopsis*	*Arenipses*	*Arge*	*Argyrotaenia*	*Arhyssus*	*Aristobia*
Arnoldiola	*Arocatus*	*Artena*	*Ascotis*	*Asiacornococcus*	*Aspidiella*	*Aspidiotus*
Asterobemisia	*Asymmetrasca*	*Athalia*	*Atherigona*	*Athlia*	*Atrachya*	*Atractomorpha*
Atta	*Attacus*	*Aulacaspis*	*Aulacophora*	*Aulacorthum*	*Aularches*	*Austroagallia*
Autographa	*Bacchisa*	*Bactrocera*	*Bagrada*	*Baileyothrips*	*Balanococcus*	*Balclutha*
Basilepta	*Batocera*	*Batrachedra*	*Belionota*	*Bemisia*	*Bephratelloides*	*Biston*
Blapstinus	*Blastobasis*	*Blastodacna*	*Blastopsylla*	*Blenina*	*Bombyx*	*Bostrychopsis*
Bothrogonia	*Brachmia*	*Brachycaudus*	*Brachycyttarus*	*Brevicoryne*	*Brontispa*	*Bruchidius*
Bruggmanniella	*Busseola*	*Bustomus*	*Buzura*	*Cacoecimorpha*	*Cacopsylla*	*Cactoblastis*
Cactophagus	*Cagosima*	*Calguia*	*Callideriphus*	*Callipterinella*	*Callosobruchus*	*Calyptocephala*
Calyptra	*Cameraria*	*Camptopus*	*Campylomma*	*Canephora*	*Cantao*	*Capnodis*
Carausius	*Carbula*	*Carmenta*	*Carposina*	*Cassida*	*Catocala*	*Caulophilus*
Cavariella	*Cephonodes*	*Cerace*	*Ceratitis*	*Ceratothripoides*	*Ceratothrips*	*Cerconota*
Cerococcus	*Ceroplastes*	*Cervaphis*	*Cetonia*	*Chaetanaphothrips*	*Chaetocnema*	*Chaetococcus*
Chalcoscelides	*Chalioides*	*Chauliognathus*	*Chelymorpha*	*Cheromettia*	*Chilecomadia*	*Chileulia*
Chilo	*Chloroclystis*	*Chlorophorus*	*Chlumetia*	*Chondracris*	*Choristoneura*	*Chromatomyia*
Chrysobothris	*Chrysodeixis*	*Chrysolina*	*Chrysomela*	*Chrysomphalus*	*Cicadella*	*Cicadulina*
Cifuna	*Cixius*	*Cleoporus*	*Cleora*	*Clepsis*	*Cletus*	*Coccus*
Coelonia	*Comotia*	*Conchaspis*	*Conoderus*	*Conogethes*	*Conopomorpha*	*Conotrachelus*
Contarinia	*Copitarsia*	*Coptotermes*	*Corylobium*	*Cosmopolites*	*Costelytra*	*Crisicoccus*
Crocidolomia	*Crocidosema*	*Croesia*	*Cryptoblabes*	*Cryptococcus*	*Cryptophlebia*	*Cryptorhynchus*
Cryptotermes	*Ctenarytaina*	*Curculio*	*Cydia*	*Dacus*	*Danothrips*	*Darna*
Delia	*Dendrolimus*	*Dendrothripoides*	*Deudorix*	*Diabolocatantops*	*Diabrotica*	*Dialeurodes*
Diaphania	*Diaphorina*	*Diaprepes*	*Diaspidiotus*	*Diatraea*	*Dinaspis*	*Diocalandra*
Diolcus	*Dischista*	*Diuraphis*	*Dolichothrips*	*Dolycoris*	*Drepanococcus*	*Dryocosmus*
Duplachionaspis	*Duponchelia*	*Dysmicoccus*	*Dyspessa*	*Earias*	*Ecdytolopha*	*Ectomyelois*
Elixothrips	*Elytroteinus*	*Empoasca*	*Enarmonia*	*Epichoristodes*	*Epiphyas*	*Erannis*
Ercheia	*Erebus*	*Eremnus*	*Eriococcus*	*Erosomyia*	*Eucalyptolyma*	*Eucarazzia*
Eudocima	*Eumeta*	*Euproctis*	*Eurygaster*	*Eurytoma*	*Euscepes*	*Exallomochlus*
Exosoma	*Eysarcoris*	*Ferrisia*	*Fiorinia*	*Frankliniella*	*Fulmekiola*	*Furcaspis*
Geniocremnus	*Glycaspis*	*Grammophorus*	*Grapholita*	*Greenidea*	*Gryllus*	*Gypsonoma*
Hadula	*Halyomorpha*	*Haplothrips*	*Haritalodes*	*Heilipus*	*Helicoverpa*	*Heliophisma*

Heliothis	*Hellula*	*Helopeltis*	*Hemiberlesia*	*Hendecasis*	*Hesperophanes*	*Heteronychus*
Heteropsylla	*Homalodisca*	*Homonopsis*	*Howardia*	*Hyadaphis*	*Hydrellia*	*Hylobius*
Hypogeococcus	*Hypothenemus*	*Hypsipyla*	*Hypurus*	*Icerya*	*Inachis*	*Insignorthezia*
Ischnopterapion	*Isotenes*	*Kerria*	*Lagria*	*Laminicoccus*	*Lampides*	*Laodelphax*
Leguminivora	*Lema*	*Lepidosaphes*	*Leptocorisa*	*Leptocybe*	*Leptodictya*	*Leucaspis*
Leucinodes	*Leucoma*	*Leucoptera*	*Ligyrus*	*Lilioceris*	*Liriomyza*	*Lobesia*
Lobometopon	*Lygocoris*	*Lymantria*	*Macchiademus*	*Maconellicoccus*	*Macrolophus*	*Macrosiphum*
Mamestra	*Maruca*	*Mayetiola*	*Megalurothrips*	*Meibomeus*	*Melanagromyza*	*Melanaspis*
Mesohomotoma	*Metamasius*	*Metopoplax*	*Miscanthicoccus*	*Monochamus*	*Morganella*	*Myllocerus*
Mythimna	*Myzus*	*Nasonovia*	*Nasutitermes*	*Naupactus*	*Neohydatothrips*	*Neoleucinodes*
Neomaskellia	*Neosilba*	*Neotermes*	*Neotoxoptera*	*Nesidiocoris*	*Nezara*	*Nilaparvata*
Nipaecoccus	*Noctua*	*Nysius*	*Odinia*	*Odonaspis*	*Oebalus*	*Oemona*
Omphisa	*Opogona*	*Opuntiaspis*	*Oraesia*	*Orchamoplatus*	*Orthezia*	*Oryctes*
Ostrinia	*Othreis*	*Otiorhynchus*	*Oxycarenus*	*Pachnoda*	*Pachymerus*	*Palaeopus*
Palmaspis	*Palmicultor*	*Pammene*	*Pandemis*	*Papilio*	*Parabemisia*	*Paracoccus*
Paraleyrodes	*Parandrita*	*Parapristina*	*Paraselenaspidus*	*Paratachardina*	*Parlatoria*	*Patchiella*
Pectinophora	*Pericyma*	*Perkinsiella*	*Phenacoccus*	*Phidotricha*	*Philephedra*	*Philotrypesis*
Phlyctinus	*Phoracantha*	*Phthorimaea*	*Phycita*	*Phyllocnistis*	*Phyllophaga*	*Pieris*
Pinnaspis	*Planchonia*	*Planococcus*	*Platypus*	*Plutella*	*Poliaspoides*	*Ponticulothrips*
Popillia	*Porotermes*	*Prays*	*Prepops*	*Priobium*	*Proeulia*	*Profenusa*
Prosapia	*Prostephanus*	*Protopulvinaria*	*Pryeria*	*Psacothea*	*Pseudanaphothrips*	*Pseudaonidia*
Pseudaulacaspis	*Pseudococcus*	*Psylliodes*	*Pteromalus*	*Pulvinaria*	*Puto*	*Pyrilla*
Pyropotosia	*Pyrrhalta*	*Pyrrhocoris*	*Quadrastichus*	*Rastrococcus*	*Retithrips*	*Rhabdoscelus*
Rhagoletis	*Rhaphigaster*	*Rhinacloa*	*Rhipiphorothrips*	*Rhizoecus*	*Rhopalosiphum*	*Rhynchophorus*
Rhyparochromus	*Richia*	*Russelliana*	*Sceliodes*	*Scepticus*	*Schistocerca*	*Sciocoris*
Scirtothrips	*Scolypopa*	*Scolytus*	*Scotinophara*	*Serrodes*	*Sesamia*	*Silba*
Silvanus	*Singhiella*	*Sinoxylon*	*Sipha*	*Siphanta*	*Siphoninus*	*Sitobion*
Sminthurus	*Solenopsis*	*Sphaeroderma*	*Spodoptera*	*Stathmopoda*	*Stegobium*	*Stenchaetothrips*
Stenoma	*Sternochetus*	*Stictococcus*	*Stromatium*	*Swammerdamia*	*Symmetrischema*	*Synanthedon*
Talponia	*Tanyrhynchus*	*Tecia*	*Telchin*	*Terellia*	*Tetraleurodes*	*Tetraneura*
Tetropium	*Thaumastocoris*	*Thaumatotibia*	*Thaumetopoea*	*Thrips*	*Thysanofiorinia*	*Thysanoplusia*
Tibraca	*Tipula*	*Tomicus*	*Tortrix*	*Toxoptera*	*Toxotrypana*	*Trachylepidia*
Tremex	*Trichoplusia*	*Trioza*	*Trogoderma*	*Trypodendron*	*Tuta*	*Tychius*
Udinia	*Unaspis*	*Uraba*	*Urentius*	*Uroleucon*	*Vinsonia*	*Wasmannia*
Xiphydria	*Xyleborus*	*Xylopsocus*	*Xylosandrus*	*Xylotrechus*	*Yponomeuta*	*Zaprionus*
Zonocerus						
**MITES**						
Aceria	*Aculops*	*Allonychus*	*Amphitetranychus*	*Aplonobia*	*Aponychus*	*Atrichoproctus*

Brevipalpus	*Bryobia*	*Cecidophyopsis*	*Cenopalpus*	*Cisaberoptus*	*Eotetranychus*	*Eriophyes*
Eutetranychus	*Halotydeus*	*Meyernychus*	*Mononychellus*	*Neotetranychus*	*Oligonychus*	*Panonychus*
Paraponychus	*Petrobia*	*Polyphagotarsonemus*	*Porcupinychus*	*Pseudobryobia*	*Raoiella*	*Schizotetranychus*
Spinacus	*Steneotarsonemus*	*Tegolophus*	*Tenuipalpus*	*Tetranychus*		
**MOLLUSKS**						
Achatina	*Archachatina*	*Arion*	*Beckianum*	*Bulinus*	*Cantareus*	*Cernuella*
Cochlicella	*Diplosolenodes*	*Elisolimax*	*Eobania*	*Helix*	*Hygromia*	*Indoplanorbis*
Laevicaulis	*Leidyula*	*Limax*	*Limicolaria*	*Macrochlamys*	*Mariaella*	*Marisa*
Monacha	*Ovachlamys*	*Oxyloma*	*Parmarion*	*Pila*	*Pomacea*	*Sarasinula*
Succinea	*Tandonia*	*Theba*	*Veronicella*	*Zachrysia*		
**NEMATODES**						
Achlysiella	*Afenestrata*	*Anguina*	*Aphelenchoides*	*Aphelenchus*	*Bursaphelenchus*	*Ditylenchus*
Globodera	*Helicotylenchus*	*Hemicycliophora*	*Heterodera*	*Hirschmanniella*	*Hoplolaimus*	*Longidorus*
Meloidogyne	*Mesocriconema*	*Nacobbus*	*Paralongidorus*	*Paratrichodorus*	*Pratylenchus*	*Punctodera*
Radopholus	*Rotylenchulus*	*Rotylenchus*	*Scutellonema*	*Thecavermiculatus*	*Xiphinema*	*Zygotylenchus*
**PLANTS**						
Aeginetia	*Ageratina*	*Alectra*	*Alternanthera*	*Avena*	*Cephaleuros*	*Chrysopogon*
Commelina	*Cuscuta*	*Digitaria*	*Emex*	*Euphorbia*	*Hydrilla*	*Imperata*
Inula	*Ipomoea*	*Ischaemum*	*Leptochloa*	*Limnophila*	*Melastoma*	*Mikania*
Mimosa	*Monochoria*	*Orobanche*	*Oryza*	*Pennisetum*	*Polygonum*	*Pycreus*
Rottboellia	*Saccharum*	*Sagittaria*	*Salsola*	*Salvinia*	*Setaria*	*Solanum*
Striga	*Thesium*	*Tridax*	*Withania*			
**VIRUSES**						
Alfamovirus	*Apscaviroid*	*Aureusvirus*	*Babuvirus*	*Badnavirus*	*Begomovirus*	*Benyvirus*
Bigeminivirus	*Bymovirus*	*Capillovirus*	*Carlavirus*	*Carmovirus*	*Closterovirus*	*Cocadviroid*
Comovirus	*Crinivirus*	*Cucumovirus*	*Curtovirus*	*Cytorhabdovirus*	*Fijivirus*	*Furovirus*
Hostuviroid	*Ilarvirus*	*Ipomovirus*	*Luteovirus*	*Monogeminivirus*	*Necrovirus*	*Nepovirus*
Nucleorhabdovirus	*Oryzavirus*	*Panicovirus*	*Pecluvirus*	*Phytoreovirus*	*Polerovirus*	*Pomovirus*
Pospiviroid	*Potexvirus*	*Potyvirus*	*Rymovirus*	*Sobemovirus*	*Tenuivirus*	*Tobamovirus*
Tombusvirus	*Torradovirus*	*Tospovirus*	*Trichovirus*	*Tymovirus*	*Umbravirus*	*Unassigned*
Unclassified	*Waikavirus*					

Table S3. Kolmogorov-Smirnov Tests to compare empirical cumulative distribution functions for number of known hosts for each major group of pests.

	Bacteria	Fungi	Oomycetes	Insects	Mites	Mollusks	Nematodes	Viruses	Plants
Bacteria		0.4925	0.6329	**0.0206**	0.1400	0.0893	0.2224	**0.0018**	**0.03513**
Fungi	0.1288		0.9193	**<0.0001**	**<0.0001**	**<0.0001**	**<0.0001**	**<0.0001**	**<0.0001**
Oomycetes	0.1409	0.0804		**<0.0001**	**0.0089**	**0.0014**	**0.0036**	**<0.0001**	**0.0015**
Insects	0.2159	0.3242	0.3128		0.5488	0.8786	0.6483	0.1502	0.9861
Mites	0.1917	0.283	0.2591	0.0779		0.7481	0.9247	0.2104	0.7915
Mollusks	0.2538	0.3741	0.3752	0.0900	0.1186		0.4310	0.9785	0.9974
Nematodes	0.1777	0.284	0.2863	0.0762	0.0734	0.1556		0.2445	0.8814
Viruses	0.3057	0.4099	0.3900	0.1052	0.1334	0.0815	0.1333		0.8246
Plants	0.2595	0.3741	0.3313	0.0560	0.0975	0.0757	0.0901	0.0921	

The lower diagonal shows the D value and the upper value the approximate P-value. Bold indicates distributions that were significantly different.

Table S4. Descriptive moments of the number of known hosts for each major group of pests.

Moment	bacteria	fungi	oomycetes	insects	mites	mollusks	nematodes	viruses	plants
Minimum	1	1	1	1	1	1	1	1	1
1st quartile	1	1	1	1	1	2	1	2	1.5
Median	2	1	2	4	4	5	4	5	5
Mean	6.308	8.118	4.295	10.33	9.975	9.6	7.79	7.333	8.465
3rd quartile	7.25	4	4	12	10	10	12	9	11
Maximum	67	184	54	109	110	84	49	43	56

Table S5. Descriptive moments of the number of known pests from each major group on the 210 host genera.

Moment	AllPests	bacteria	fungi	oomycetes	insects	mites	mollusks	nematodes	viruses	plants
Minimum	7	0	0	0	1	0	0	0	0	0
1st quartile	37	0	4.25	0	21	2	0	0	0	0
Median	52	1	7	1	32	4	1	1	1	1
Mean	72.99	1.562	8.195	1.248	42.8	5.652	2.057	3.895	4.714	2.862
3rd quartile	92.25	2	11	2	51.75	8	3	5	5	4
Maximum	426	16	59	14	234	27	21	45	64	30
Max possible	1670	52	212	61	870	119	45	105	135	71

Table S6. Coefficients of logistic regressions of phylogenetic distance, number of known hosts, and the interaction, on host sharing.

Pest Group	Coefficients β_0	Coefficients β_1	Coefficients β_2	Coefficients β_3	95% Confidence Intervals β_0	95% Confidence Intervals β_1	95% Confidence Intervals β_2	95% Confidence Intervals β_3
Bacteria	5.2051	-4.2204	-0.0585	0.0533	3.5321 : 6.3572	-4.7416 : -3.4835	-0.1771 : 0.1552	-0.0351 : 0.1043
Fungi	5.8396	-4.4362	0.0655	-0.0080	4.6278 : 6.7301	-4.8305 : -3.9361	-0.0333 : 0.1862	-0.0581 : 0.0339
Oomycetes	3.2735	-3.5576	0.0784	0.0015	-0.0278 : 4.6686	-4.1705 : -2.2712	-0.1244 : 0.9712	-0.3601 : 0.0876
Insects	4.2176	-3.5346	0.0059	0.0201	3.6914 : 4.7140	-3.7461 : -3.3064	-0.0256 : 0.0507	0.0011 : 0.0336
Mites	2.5842	-2.8515	0.0476	-0.0013	1.5056 : 3.5927	-3.2868 : -2.3731	-0.0388 : 0.1433	-0.0413 : 0.0354
Mollusks	-1.0676	-1.2140	0.0315	0.0068	-5.1800 : 0.9175	-2.0593 : 0.4888	-0.0489 : 0.4080	-0.1471 : 0.0411
Nematodes	4.5541	-3.8947	-0.0948	0.0789	2.9370 : 5.5028	-4.3069 : -3.2069	-0.1925 : 0.0762	0.0068 : 0.1208
Viruses	8.4099	-5.6770	0.0905	0.0024	7.0665 : 9.4959	-6.1504 : -5.1201	-0.0531 : 0.2830	-0.0771 : 0.0648
Plants	1.8882	-2.6429	0.0998	-0.0115	0.4730 : 3.0591	-3.1429 : -2.0661	-0.0366 : 0.2805	-0.0845 : 0.0466

Coefficients of logistic regressions (median and 95% confidence intervals) of phylogenetic signal in host sharing with two independent variables (phylogenetic distance between source host genus and target host genus (coefficient β_1) and the number of known hosts for the pest (coefficient β_2)) and their interactions (coefficient β_3). The dependent variable was whether the target host genus was known to be susceptible (S) to a pest from the source host genus. The regression takes the form of $\text{logit}(S)= \beta_0 + \beta_1 * \log_{10}(PD +1) + \beta_2*(\text{Number of known hosts}) + \beta_3*[(\log_{10}(PD +1)) *(\text{Number of known hosts})]$. Number of known hosts and the interaction term (β_3) were only significant for insects and nematodes (95%CIs overlapped zero in others) and the number of hosts was not significant as a main term in any of the models. As such, only main effects, and not the interaction term, were included in the models presented in the text (Table 2, Figure 3).
