# Supplementary figures and images for: Evolutionary tools for phytosanitary risk analysis: phylogenetic signal as a predictor of host range of plant pests and pathogens
Source: Evol Appl. 2012 May 3;5(8):869–78. doi: 10.1111/j.1752-4571.2012.00265.x (PMC3552404; doi:10.1111/j.1752-4571.2012.00265.x)

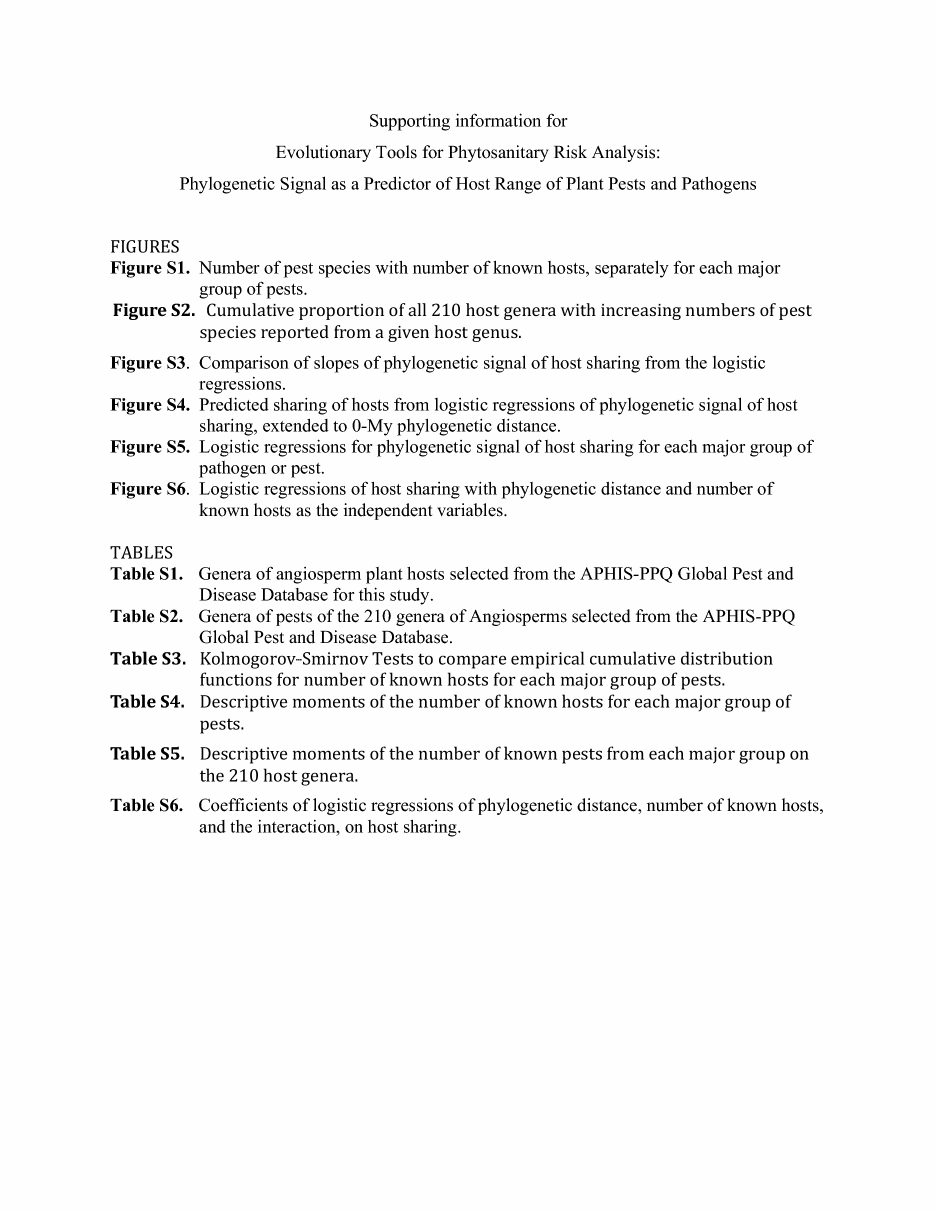

Supplement: Supplementary file 4 [file eva0005-0869-SD4.png]
